# Profiling of *Bacillus thuringiensis* Biopesticides: A High-Resolution LC–MS/MS Approach for Subspecies Identification and Protein Quantification

**DOI:** 10.1021/acs.jafc.5c16915

**Published:** 2026-07-01

**Authors:** Sara Schlachter, Beatrix Stessl, Martina Ludewig, Elisabeth Reiter, Irmengard Strnad, Evelyn Rampler, Stefano D’Amico

**Affiliations:** † Institute for Animal Nutrition and Feed, 31190 Austrian Agency for Health and Food Safety, Spargelfeldstraße 191, 1220 Vienna, Austria; ‡ Unit of Food Microbiology, Centre for Food Science and Public Veterinary Health, Clinical Department for Farm Animals and Food Systems Safety, University of Veterinary Medicine Vienna, Veterinärplatz 1, 1210 Vienna, Austria; § Department of Analytical Chemistry, Faculty of Chemistry, 27258 University of Vienna, Währinger Straße 38, 1090 Vienna, Austria

**Keywords:** proteomics, insecticidal proteins, parallel reaction monitoring, Bt spp. kurstaki, aizawai, israelensis

## Abstract

The widespread use of *Bacillus thuringiensis* (*Bt*)-based biopesticides in organic farming presents challenges for limited data on insecticidal protein concentrations in treated samples and the environment after the application of *Bt* biopesticides. To address this, an LC-HR-MS/MS-PRM method for the identification and quantification of crystal proteins from *Bt* subspecies *kurstaki*, *aizawai*, and *israelensis* was developed. Marker peptides were selected based on untargeted analyses and validated for subspecies differentiation and quantification using stable-isotope-labeled (SIL) standards. The method demonstrated high sensitivity (ng/g range), repeatability precision, and selectivity across various matrices. Application to basil plants treated with *Bt* biopesticides confirmed accurate subspecies identification and protein quantification. This approach supports product quality control and environmental safety by providing qualitative and quantitative data on *Bt* toxins in treated samples depending on the applied *Bt* biopesticide. It offers a robust analytical tool that addresses key limitations of current approaches.

## Graphical abstract

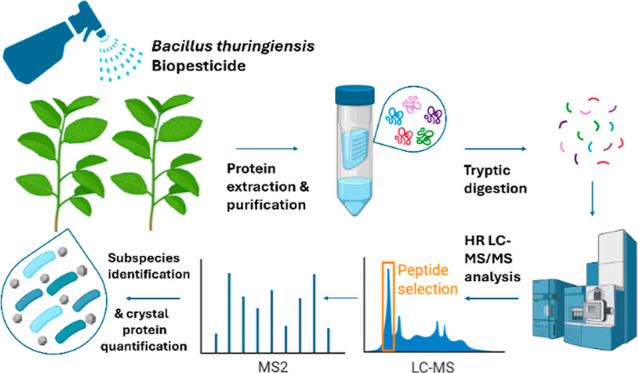

## 1 Introduction

*Bacillus thuringiensis* (*Bt*) is a Gram-positive bacterium belonging to the *Bacillus cereus* group, renowned for its ability to produce insecticidal proteins.^1^ Products containing *Bt* are the most widely used biopesticides for insect pest control due to their effectiveness, high specificity toward target organisms, and harmlessness to nontarget organisms and the environment.^2,3^ The insecticidal proteins produced by *Bt* are classified into two groups based on their production phase: Cry and Cyt proteins, which are formed in parasporal inclusions during the stationary growth phase, and Vip and Sip proteins, which are secreted during the vegetative growth phase. These insecticidal proteins vary in their biological activity and target organisms. Cry and Cyt proteins, also known as δ-endotoxins, are primarily effective against insects from the orders *Diptera*, *Coleoptera,* and *Lepidoptera*.^4^ Different models have been proposed to explain the mechanism of action of *Bt* crystal toxins. The classical model, known as the sequential union model, assumes that the crystals are inactive protoxins and become toxic only after ingestion by insects. Once activated, the proteins bind to specific receptors on the insects’ gut cell membranes, forming pores in the membrane. These pores disrupt the membrane integrity, leading to osmotic imbalance, cell lysis, total paralysis, and finally the insects’ death.^5,6^

Recent EFSA peer reviews for different *Bt* strains and subspecies concluded general safety for consumers and the environment of *Bt* biopesticides, when used as authorized.^7–10^ However, EFSA also identified data gaps addressing (i) potential risk for human health through nondietary exposure for residents, bystanders, or workers and (ii) the risk to nontarget terrestrial and aquatic organisms due to insufficient hazard characterization and exposure information in the environment after the applications of *Bt* biopesticides.^7,9^ To evaluate the environmental exposition, it is necessary to investigate the insecticidal activity depending on the *Bt* subspecies and strain by quantifying Cry protein contents. For this purpose, a highly sensitive and specific analytical method is required.

DNA-based technologies, such as polymerase chain reaction and genome sequencing enable the characterization of *Bt*-specific *cry* and *cyt* gene contents and can differentiate between *Bt* subspecies and strains.^11,12^ However, these methods reach a limitation when it comes to the prediction of insecticidal activity, which is determined by gene expression. Therefore, the identification and quantification of insecticidal proteins are essential for assessing the toxic potential of *Bt* biopesticides.^13^ Quantification at the protein level was attempted using reverse-phase high-performance liquid chromatography (RP-HPLC)^14^ and ion-exchange liquid chromatography.^15^ However, these applications are unable to distinguish between the different insecticidal proteins due to their similar amino acid sequences. Enzyme-linked immunosorbent assays (ELISA) offer greater specificity^16,17^ but typically target only a limited number of proteins or only one. Recent reviews have summarized the state of analytical approaches for *Bt* Cry protein detection and quantification, highlighting immunoassay-based methods as the current standard while also emphasizing limitations related to specificity due to cross-reactions among very similar Cry proteins, matrix effects, and restricted multiplexing capability.^18^ Additionally, recent reviews focusing on the analysis of bacterial and food-relevant toxins have highlighted liquid chromatography–tandem mass spectrometry (LC–MS/MS)-based approaches as powerful tools for sensitive and specific detection and quantification of protein toxins in complex matrices while also discussing current challenges related to sample preparation, sensitivity, and standardization.^19^ LC–MS/MS was also used to analyze different *Bt* subspecies and strains, though most studies focus on protein identification rather than quantification.^20–22^

LC-MS-based proteomics approaches enable the quantification of complex protein mixtures using highly specific proteolytic peptides, offering a powerful analytical tool for trace analysis.^23^ The study of Caballero et al.^24^ is the first to develop and validate an LC–MS/MS-multiple reaction monitoring method for identifying and quantifying insecticidal crystal proteins in various *Bt* products. However, their study was limited to a single strain for each authorized *Bt* subspecies and did not allow the identification of subspecies based on the selected peptide sequences. Moreover, the method did not include the analysis of samples treated with *Bt*-containing biopesticides. The aim of this study was to develop a targeted high-resolution LC–MS/MS-parallel reaction monitoring (PRM) method to overcome the mentioned limitations. It is the first attempt to combine identification and quantification in one analytical run by using (1) unique marker peptides for the identification of the authorized *Bt* spp. *kurstaki*, *aizawai*, and *israelensis* and (2) including shared marker peptides for the absolute quantification of insecticidal crystal proteins using stable isotope-labeled (SIL) peptides. Furthermore, an optimized sample preparation workflow, including enrichment of the analytes, was developed to enable the analysis of both *Bt*-containing products and samples treated with *Bt* biopesticides with high sensitivity.

## 2 Material and Methods

### 2.1 Chemicals and Sample Material

Acetonitrile and methanol in LC–MS-grade quality were purchased from Honeywell (Morristown, New Jersey, USA). Iodoacetamide (≥99%), trifluoroacetic acid, 1,4-dithiothreitol (DTT), ammonium bicarbonate (≥99% for LC–MS), tris­(hydroxymethyl)-aminomethane (TRIS), bovine serum albumin (BSA) (≥98%), and apomyoglobin from equine skeletal muscle (≥97%, 54 nmol) were purchased from Sigma-Aldrich (Bornem, Belgium). Urea, hydrochloric acid (32%), and formic acid (≥99% for LC–MS) were purchased from VWR (Darmstadt, Germany). RapiGestTM SF Surfactant was purchased from Waters (Milford, Massachusetts, USA). Pierce Trypsin Protease in MS grade was purchased from Thermo Fisher (Dreieich, Germany). ROTIQuant 5x concentrate was purchased from Carl Roth (Karlsruhe, Germany). SIL peptide standards were obtained from GenScript (Rijswijk, Netherlands). For all experiments, ultrahigh quality (UHQ) water, type 1 (conductibility of 0.055 μS/cm and filtered by 0.2 μm sieve) was used. The biopesticides LepinoxPlus (Kwizda Agro GmbH, Vienna, Austria), Xentari RaupenFrei (W. Neudorff GmbH KG, Emmerthal, Germany), VectoBac (Valent BioSciences, Illinois, USA), DiPelDF (Certis Belchim B. V., Gleisdorf, Austria), Gnatrol SC (Biofa GmbH, Münsingen, Germany), Neudomück (Progema GmbH, Aerzen, Germany), Solabiol Zünsler-& Raupenfrei (SBM, Ecully, France), and FlorBac (Certis Belchim B. V., Gleisdorf, Austria) were purchased from the corresponding online retailers. Dried cultures DSM 350, DSM 5724, DSM 5725, DSM 6110, DSM 6070, DSM 6099, DSM 5019, and DSM 12001 were purchased from Leibniz Institute DSMZ—German Collection of Microorganisms and Cell Cultures GmbH (Braunschweig, Germany).

### 2.2 Protein Extraction and Enrichment

Two different extraction protocols and three protein enrichment strategies were evaluated and compared. For each protocol, about 2 g sample material—a mixture of Xentari RaupenFrei, LepinoxPlus, and VectoBac—was weighted into a 15 mL Falcon tube. The first extraction protocol using 2 N urea and 0.2 N TRIS (pH 9.2) was performed according to Planque et al.^25^ The second protocol using 50 mM ammonium bicarbonate and 10 mM DTT (pH 10.5) was performed according to Şahin et al.^26^ In both cases, 20 mL of the extraction reagent was added to each sample. Protein extracts were filtered, first using a syringe filter of 0.8 μm (PES, Pall Cooperation, Vienna, Austria) and subsequently with 0.45 μm membrane (regenerated cellulose—RC, 0.45 μm, Phenomenex, Aschaffenburg, Germany) prior to enrichment. Three enrichment strategies were assessed: Sephadex G-25 PD-10 desalting columns (Cytiva, Wilmington, USA), Vivaspin 6 5K, and Vivaspin 6 10 K centrifugal concentrators (Sartorius AG, Göttingen, Germany). Each method was applied to 5 mL of protein extract following the corresponding manufacturer’s instructions,^27,28^ resulting in a final volume of approximately 0.5 mL. Protein content was determined by the Bradford assay^29^ using BSA for standard calibration. Adjusted from Geisslitz et al.,^30^ 1.2 mL of ice-cold acetone was added to 300 μL of ultrafiltrated protein extracts, and samples were incubated overnight at −20 °C for protein precipitation. After centrifugation at 4000*g* for 10 min at −4 °C, the supernatant was discarded, and the resulting pellets were washed twice with 1.2 mL of ice-cold acetone. The pellets were dried and stored at −20 °C for further sample preparation. Based on the results for the soluble protein content (Table S1), all further extractions were performed using 50 mM ammonium bicarbonate and 10 mM DTT (pH 10.5), with protein enrichment carried out using Vivaspin 6 10 K.

### 2.3 In-Solution Digestion and Solid-Phase Extraction

Adjusted from Schlachter et al.,^31^ protein pellets were directly solubilized in 300 μL of 0.05% RapiGest solution, and apomyoglobin was added to each sample as an internal standard. Subsequent steps, including reduction, alkylation, digestion, and purification of tryptic peptides, were performed according to Geisslitz et al.^30^ In detail, reduction was carried out by adding 30 μL of 100 mM DTT and incubating the samples at 60 °C for 20 min at 300 rpm using a ThermoMixer. After cooling to room temperature, alkylation was performed by adding 30 μL of 200 mM iodoacetamide and incubating in the dark for 30 min at room temperature. To remove the remaining iodoacetamide, 20 μL of 100 mM DTT was added, and samples were incubated in the dark for 10 min at room temperature. Proteins were digested using trypsin at a protein-to-enzyme ratio of around 50:1, with incubation at 37 °C and 300 rpm for 17 h. Digestion was stopped the next day by adding 10–20 μL of 5% TFA to a pH ≥ 2, and the digested extracts were centrifuged (8000 rpm for 3 min) to remove insoluble polymers. Peptides were then purified by SPE cleanup using self-packed stage tips containing six layers of SDB-XC reversed-phase Empore extraction disks (3 M Deutschland GmbH, Neuss, Germany). The stage tips were equilibrated with methanol and washed with 2.5% ACN in water containing 0.1% TFA (v/v). Peptide extracts were loaded onto stage tips and washed twice. Peptides were eluted with 60% ACN in water and 0.1% TFA (v/v), dried under vacuum, and reconstituted in 10% ACN with 0.1% FA (v/v) in water for LC–MS analysis. SIL standards were added immediately prior to measurements.

### 2.4 Purification of Proteins from *Bt* Isolates

The *Bt* isolates (see products, Section 2.1) were stored at −70 °C in cryocultures (Brain–Heart–Infusion broth, Biokar Diagnostics, Beauvais Cedex, France with 25% glycerol) in the strain collection of the Center for Food Safety and Public Veterinary Health until the start of the experiment. The isolates were plated on Tryptone Casein Soy Agar with 0.6% yeast extract (TSAY, Biokar Diagnostics, Beauvais Cedex, France) and incubated at 30 °C for 24 h. From these cultures, a 10 μL loop of colony material was transferred into 5 mL of Tryptone Soy Broth (TSBY, Oxoid Ltd., Basingstoke, Hampshire, England). The broth was homogenized and incubated on a shaker at 120 rpm and 30 °C for 24 h. Subsequently, 100 μL of each liquid culture was distributed onto five TSAY agar plates and incubated at 30 °C for 72 h. The obtained bacterial lawn was collected using a 10 μL loop and suspended in 20 mL of Ringer’s solution (B. Braun, Melsungen, Germany). The suspension was vortexed and centrifuged at 4000 rpm for 10 min at 4 °C. The supernatant was carefully removed and discarded using a pipet. Protein extraction and purification were performed following the protocol described by Mounsef et al.^32^ with some modifications. The resulting pellet was washed twice with 1 M NaCl containing 0.01% Triton X-100 (Merck KGaA, Darmstadt, Germany). Next, a 10% hexane–NaCl–Triton X solution was added, and the pellet was sonicated in an ultrasonic bath at 350 W for 10 min and centrifuged at 4000 rpm for 10 min at 4 °C. This step was repeated twice. The obtained pellet was subjected to a thorough washing process and stored at −20 °C until further preparation. For proteomic analysis, protein pellets were thawed for 30 min at room temperature, and proteins were extracted from the pellets as described in Section 2.2, followed by digestion and purification according to the procedure outlined in Section 2.3.

### 2.5 Application of *Bt*-Products

For the method development and assessment of matrix effects, 5 g granulates of LepinoxPlus, Xentari RaupenFrei, and VectoBac, as well as 5 mL of Solabiol Zünsler-& Raupenfrei, were suspended in 500 mL of tap water. These preparations were applied to basil, mint, parsley, and lovage plants, which were incubated for 5 days indoors at room temperature to prevent any contamination. Leaf samples were collected into 50 mL Falcon tubes, and sample preparation was performed as described in Sections 2.2 and 2.3. To evaluate the performance of the final PRM method under realistic application conditions, all four biopesticides were applied to basil plants following the manufacturer’s instructions. In detail, the following concentrations were used to prepare 50 mL suspensions with tap water: LepinoxPlus—10 g in 5–15 L for 100 m^2^ (recommended for herbs); Solabiol Zünsler-& Raupenfrei—1 mL in 1 L for 8–17 m^2^ (recommended for ornamental plants; no indication for herbs); Xentari RaupenFrei—3 g in 1.8 L for 15–30 m^2^ (recommended for ornamental plants; no indication for herbs); VectoBac—3 g in 50 L (recommended for mosquito larva; no indication for herbs). Approximately 25 mL of each suspension was applied to two basil plants per product and incubated indoors at room temperature for 5 days. After incubation, leaves from basil plants for each product were weighed (10 g) into 50 mL Falcon tubes, and sample preparation was performed as described in Sections 2.2 and 2.3.

### 2.6 LC–QTOF–MS Analysis

Separation of digested peptides was performed by a Waters UPLC-System ACQUITY Bio H-Class equipped with a CSH C18 (1 mm × 150 mm, 1.7 μm) column. For all acquisition modes, identical LC conditions (gradient with water containing 0.1% FA (A) and 98% ACN in water containing 0.1% FA (B)) were applied. Detection was performed with a Xevo G2 XS QTOF mass spectrometer from Waters. Detailed setup of gradient separation and mass spectrometry settings are listed in Table S2. Data-independent acquisition (DIA) was performed in SONAR mode using a 35 Da isolation window according to Schlachter et al.^31^

### 2.7 Bioinformatic Tools and Statistics

Basic local alignment sequence tool (BLAST) was performed using UniProtKB reference proteomes and Swiss-Prot database. Untargeted data-dependent acquisition and DIA data were processed using ProteinLynx Global Server (PLGS, Waters; version 3.0.3). DIA-based protein identification was conducted using imported *Bt* databases from UniProtKB within PLGS. See Tables S3 and S4 for detailed information. To develop the targeted PRM method and optimize collision energies for selected peptide markers, the MassLynx–Skyline interface (version 4.2) was employed. Targeted data were evaluated by using Skyline (version 24.1.0.199) and TargetLynx (version 4.2). Statistical analyses were performed by using IBM SPSS Statistics (version 26.0.0.1).

### 2.8 Absolute Quantification Using SIL

Peptide standards were dissolved either in UHQ water or 50% ACN depending on their individual solubility, to achieve a final concentration of 4 mg/mL for each stock solution. Two mixtures were prepared: one containing all light peptide standards and another containing the corresponding SIL standards, each at a final concentration of 0.4 mg/mL. These mixtures were further diluted using 10% ACN with 0.1% FA as needed. Calibration was performed across a range of 3.35 fmol/μL to 42950 fmol/μL (corresponds to 11.7 ng/g to 150009 ng/g), using at least five calibration points within this range, depending on the individual performance of each peptide. The SIL concentration level was selected to ensure good detectability. All calibrations were conducted in duplicate. For data evaluation, the light-to-heavy ratios of the two most abundant transitions were calculated using Skyline (version 24.1.0.199). Detailed information and calibration curves are provided in Table S5.

## 3 Results and Discussion

### 3.1 Identification of Crystal Proteins for *Bt* spp. *kurstaki*, *aizawai*, and *israelensis*

The search using UniProtKB yielded six pesticidal crystal proteins associated with *Bt* spp. *israelensis*, including four reviewed and two unreviewed entries. For *spp. kurstaki*, nine reviewed pesticidal crystal proteins were found, while *spp. aizawai* yielded eight reviewed entries. Untargeted DIA analyses of crystal protein pellets (Section 2.4), as well as of applied commercial products on various herbs (Section 2.5), enabled the identification of predominant pesticidal crystal proteins across the three *Bt* subspecies. Figure 1 shows the general workflow for protein identification and development of the targeted method.

**Figure 1:**
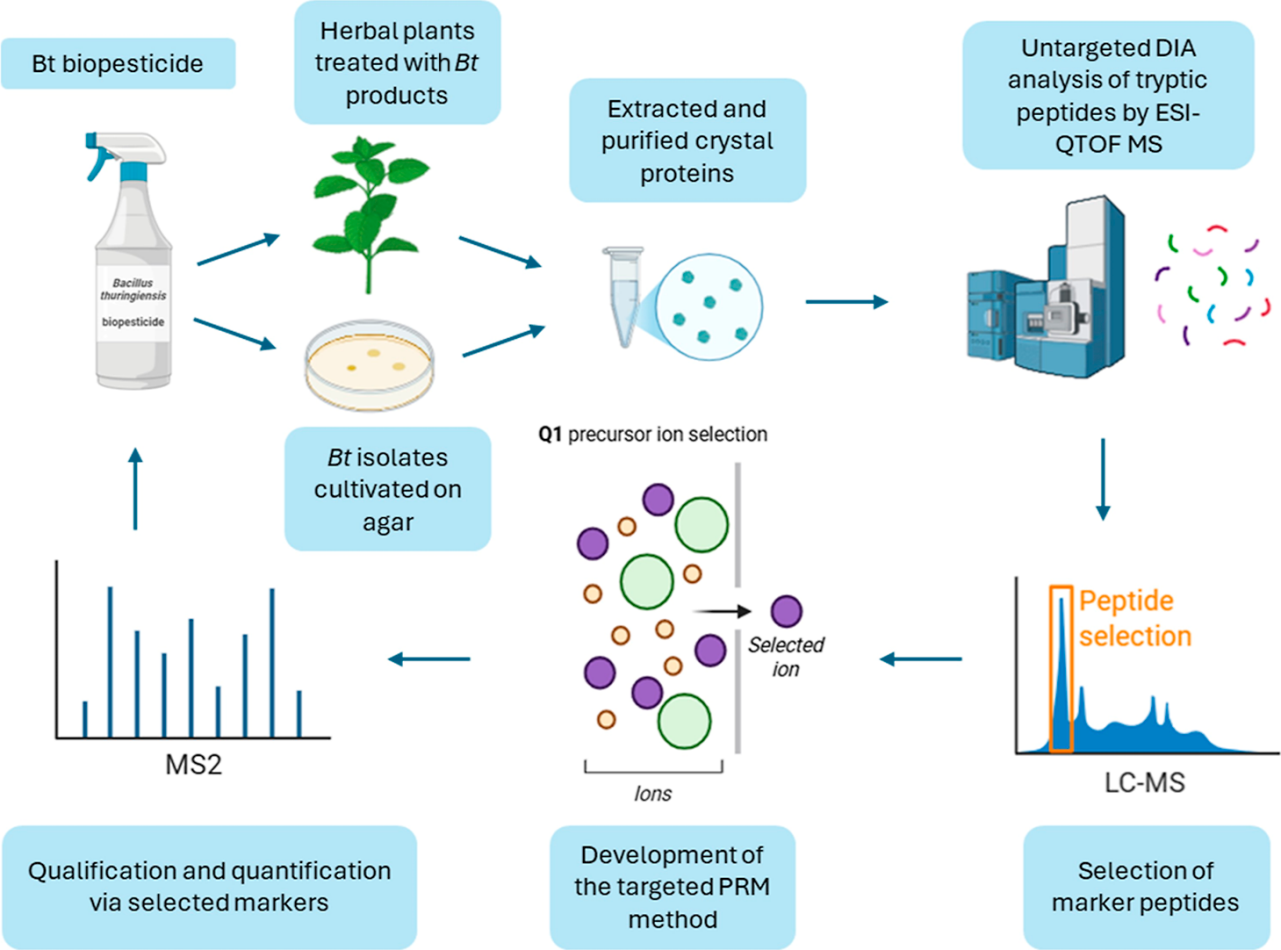
Workflow for target protein identification by DIA analyses and development of the targeted method.

No differences in protein profiles were observed between samples derived from isolated microorganisms with purified crystal proteins and those extracted directly from the treated plant material (data not shown). For *spp. israelensis*, Cry4Aa, Cry4Ba, and Cry11Aa were identified as the predominant crystal proteins, present in all three products containing this subspecies (Figure 2). These crystal proteins are described in other studies as major toxins as well.^24,33^ In the case of *spp. kurstaki*, Cry1Ac, Cry2Aa, and Cry2Ab were the major proteins detected, while Cry1Ad, Cry1Ca, and Cry1 Da were specific to *spp. aizawai*. Notably, Cry1Aa and Cry1Ab were shared between *S. kurstaki* and *S. aizawai*, indicating overlapping protein profiles. The results for identified crystal proteins are consistent with previously reported data by Caballero et al.^24^ As shown in Figure 2, no strain-level differences in the crystal protein profiles were observed between *spp. kurstaki* strains ABTS-351 and EG-2348. Also, no matrix effect was observed; all four applied *Bt* biopesticides showed identical protein profiles for basil, parsley, lovage, and mint (data not shown).

**Figure 2:**
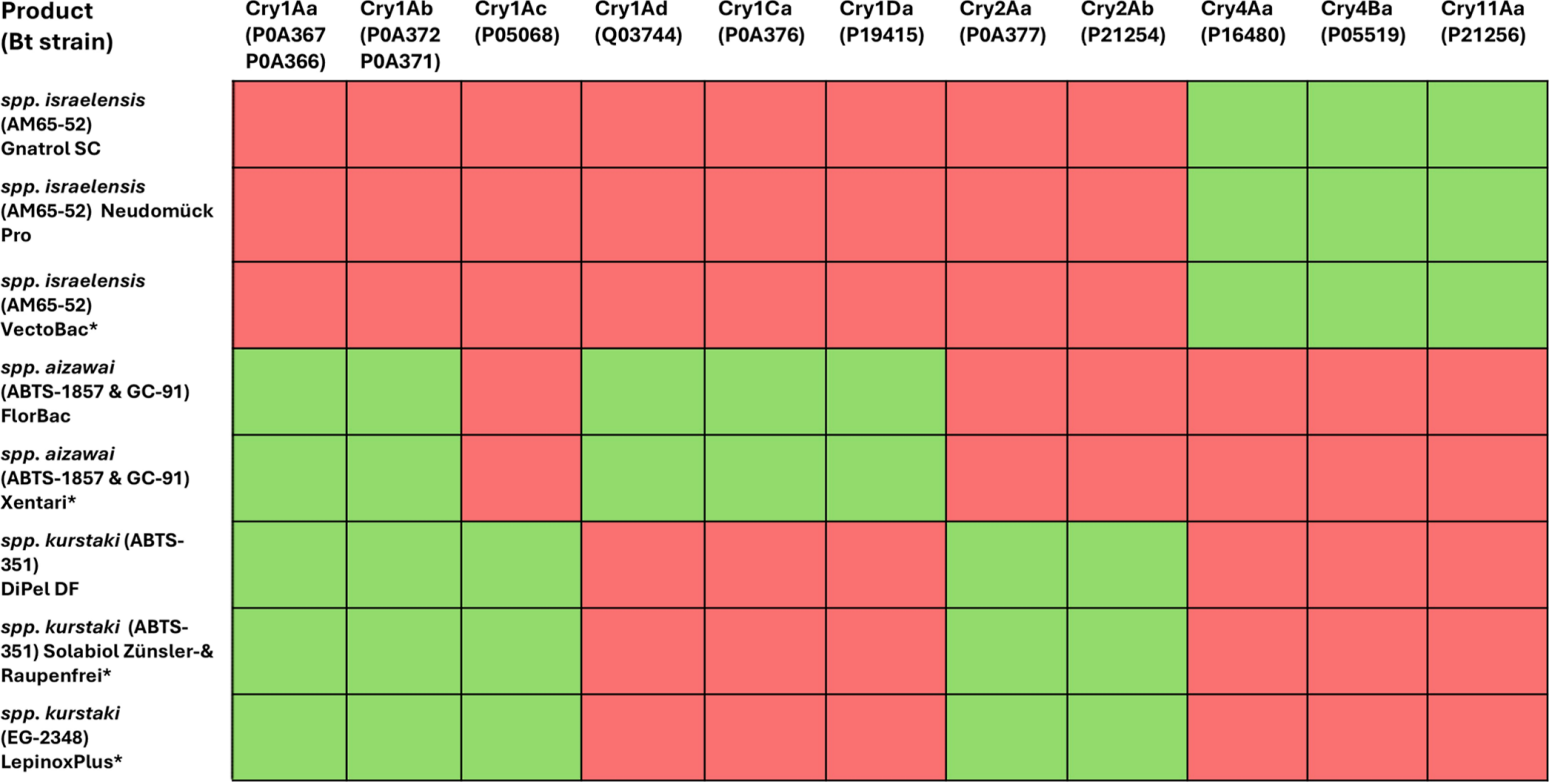
Identified crystal proteins in various commercial *Bt* biopesticides by untargeted LC–MS/MS analysis. Red: not identified; green: identified. Products marked with * were applied to different plant matrices.

### 3.2 Development of Targeted Parallel Reaction Monitoring (PRM) Method

On the basis of the untargeted DIA data, marker peptides corresponding to the identified predominant crystal proteins were selected. The objective of the developed targeted PRM method was to enable both the differentiation of the three *Bt* subspecies and the absolute quantification of the crystal protein content. The selection of suitable marker peptides is the most critical and important aspect of method development, as these peptides must fulfill specific criteria, including (i) specificity, (ii) signal intensity, (iii) sequence length, and (iv) stability.^34,35^ The use of a TOF-MS instrument also makes retention time an important parameter. If multiple analytes are measured simultaneously, TOF instruments are limited by their low duty cycles due to discontinuous ion transfer, which results in loss of sensitivity.^36^ This limitation can be partially compensated for by employing the target enhancement mode, which optimizes the ion accumulation period based on a narrow *m*/*z* range. The aim of qualitative distinction was to identify two unique peptides for each subspecies. Specificity was the primary criterion for qualitative marker peptides and was verified both in silico using UniProt database BLAST searches and experimentally. The selection of highly specific peptides was challenging due to the lack of spectral libraries for *Bt* crystal proteins as well as the high similarity of the amino acid sequence among certain proteins.^37^ For *spp. israelensis*, two peptide sequences could be selected as qualitative markers: one shared by Cry4Aa and Cry4Ba and one for Cry11Aa (Table 1). Alves et al.^38^ also identified these peptides as specific to *Bt* spp. *israelensis*. Similarly, for *spp. aizawai*, two marker peptides were identified, one for Cry1Ca and one for Cry1 Da, while for *spp. kurstaki*, only one unique marker peptide was identified for Cry1Ac (Table 1). For absolute quantification, shared peptide sequences were prioritized to enable detection and quantification of the broadest possible range of crystal proteins. The peptide sequence ELEYFPETDK was selected due to its presence in multiple Cry1 proteins from both *spp. kurstaki* and *spp. aizawai* (Table 1). Additionally, the peptide GNSNYFPDYFIR was chosen to cover Cry2Aa and Cry2Ab of *spp. kurstaki*. No shared peptide sequences were identified between *spp. israelensis* and the other subspecies; therefore, the marker peptides TVDVFPDTDR for Cry4Aa and Cry4Ba and TEVETLINQK for Cry11Aa were selected as quantitative markers which were mentioned in the literature before.^38^ To enable absolute quantification, SIL standards for the four quantitative markers were incorporated into the PRM method. Apomyoglobin from horse heart was included as an internal standard to assess the efficiency of tryptic digestion and purification. Collision energies for each marker peptide were individually optimized by using Skyline. For each peptide, five most abundant transitions were selected. To enhance the sensitivity for the quantification markers, target enhancement was applied (Table 1).

**Table 1:**
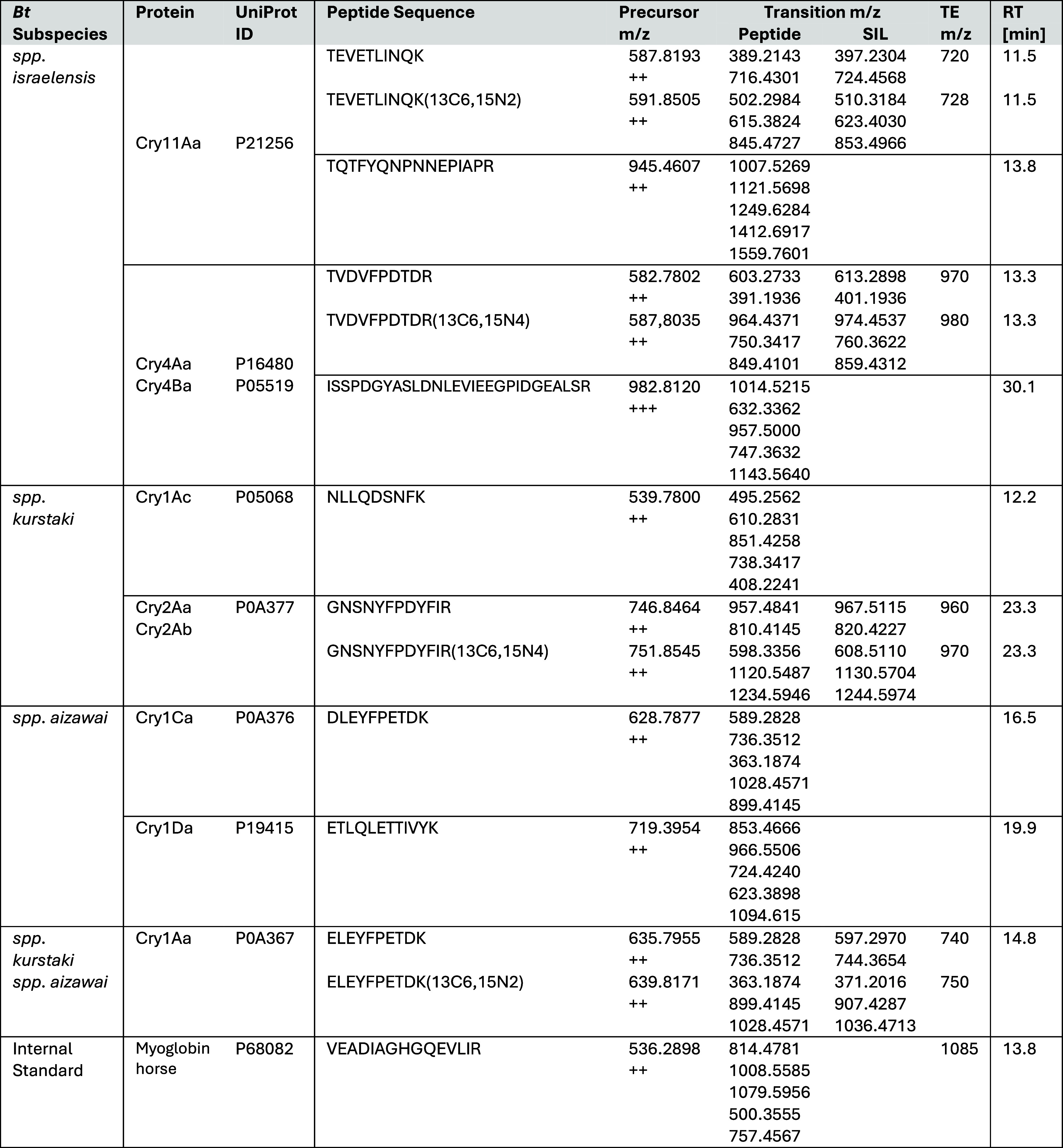
Qualitative and Quantitative Selected Marker Peptides of the Developed LC–MS/MS-PRM Method with *Bt* Subspecies, Crystal Protein, Precursor *m*/*z*, Transition *m*/*z*, Target Enhancement (TE) *m*/*z*, and Retention Time (RT)

### 3.3 Method Validation

#### 3.3.1 Evaluation of Limit of Detection (LOD) and Limit of Quantification (LOQ)

To determine the limit of detection (LOD) and limit of quantification (LOQ), peptides and their corresponding SIL standards were mixed with an analyte-free basil matrix in low concentrations, approximating the estimated LOD and LOQ levels calculated from the calibration curve.^39^ The lowest level at which a constant ratio of the transitions was still observed was used to determine the LOD and LOQ. LOD was defined as three times the standard deviation and LOQ as ten times the standard deviation of the lowest level.^39^ LOD and LOQ were verified experimentally by adding the analytes three times to the basil analyte-free matrix at the concentrations calculated for LOD and LOQ. Estimated values for the LOD and LOQ are listed in Table 2. The marker peptides were detected with high sensitivity, yielding LODs and LOQs in the nanogram per gram (ng/g) range for all peptides.

**Table 2:** Estimated Values for LOD, LOQ, and Repeatability Precision for Quantitative Peptides of the LC–MS/MS-PRM Method

				repeatability precision (Sr) (%)	
protein	peptide	LOD (ng/g)	LOQ (ng/g)	low level (40 fmol/μL)	high level (850 fmol/μL)
Cry11Aa	TEVETLINQK	0.89	2.70	0.8	0.8
Cry4Aa/Cry4Ba	TVDVFPDTDR	0.57	1.88	0.7	0.7
Cry2Aa/Cry2Ab	GNSNYFPDYFIR	10.41	34.36	3.3	4.5
Cry1Aa/Cry1Ab	ELEYFPETDK	0.17	0.65	0.9	0.7

#### 3.3.2 Evaluation of Repeatability Precision

Repeatability precision was evaluated by analyzing six replicates (*n* = 6) at two concentration levels: a low level at 40 fmol/μL and a high level at 850 fmol/μL. Quantitative marker peptides and their corresponding SIL standards were spiked into an analyte-free basil matrix. The results for repeatability (Table 2) were very good for all peptides at both concentration levels. The values for the two concentration levels were comparable and show only minor variations for all peptides, indicating high precision and reliability of the method.

#### 3.3.3 Selectivity of Marker Peptides

The selectivity of all marker peptides was evaluated in silico using UniProt database BLAST searches, as well as experimentally. Acceptance criteria for the qualitative marker peptides were defined according to Schlachter et al.^31^ A signal was considered positive if at least two transitions per precursor exhibited a signal-to-noise (S/N) ratio greater than 10, with at least one transition exceeding a ratio of 3. All three transitions had to be observed at the expected retention time at a tolerance of ±2.5%. Quantitative marker peptides were considered positive when their signal exceeded the estimated LOD (Section 3.3.1). Figure 3 presents the Skyline chromatograms of all marker peptides for the three *Bt-*based products—Xentari RaupenFrei, LepinoxPlus, and VectoBac—as well as for the blank sample containing *Bacillus mycoides*. Table S7 summarizes the selectivity results for all analyzed sample materials. The experimental results aligned with expectations across all 13 sample materials. *Bacillus mycoides* and *Lysinibacillus sphaericus* were selected as blank materials for all marker peptides. *B. mycoides* belongs also to the *B. cereus* group and is genetically closely related, while being nonpathogenic. This should allow evaluation of the method’s ability to distinguish *Bt* from other closely related species of the *B. cereus* group without posing a safety risk.^40^ *L. sphaericus* is also a Gram-positive bacterium that produces insecticidal proteins, known as Mtx and Bin toxins.^41^ Both blank materials yielded negative results for all marker peptides (Figure 3, Table S7). The qualitative marker peptides enabled accurate identification of the respective *Bt* subspecies in all three *Bt* biopesticides (Figure 3). LepinoxPlus showed a positive signal only for the qualitative marker specific to *spp. kurstaki*, while the other peptides were negative due to low signal intensity within the noise region. Similarly, Xentari RaupenFrei was positive only for the qualitative marker for *spp*. *aizawai*, and VectoBac for the two peptides specific to *spp. israelensis*. All other *Bt*-containing sample materials listed in Table S7 showed results consistent with the intended subspecies. No false-positive results were observed for *Bt spp. kumamotoensis* and *spp. galleriae* (Table S7). The quantitative marker peptide for Cry1Aa/1Ab gave positive results for LepinoxPlus and Xentari RaupenFrei (Figure 3), and notably also for sample 5 (Table S7) containing *Bt spp. galleriae*. As previously described, the quantitative markers were selected based on shared peptide sequences among various Cry1 proteins from different *Bt* subspecies to enable the most comprehensive quantification of crystal proteins. The quantitative marker for Cry2Aa/2Ab, specific for *spp*. *kurstaki*, was positive only for product LepinoxPlus, as expected. The two quantitative markers for Cry4Aa/4Ba and Cry11Aa were positive only in the product VectoBac, matching expectations. These results demonstrate the high specificity of the qualitative marker peptides for subspecies identification and the high applicability of the quantitative marker peptides for protein quantification. The use of high-resolution MS instruments, such as QTOF systems, enhances analytical confidence due to their superior mass accuracy (mass error <1 ppm using internal calibration and LockSpray system for reference correction) and resolution (sensitivity mode >30,000 fwhm). This allows highly selective compound identification and minimizes the risk of false-positive results even in complex matrices. This capability is particularly valuable in verifying marker peptide specificity.^42^ Additionally, high-resolution MS workflows are ideally suited to perform untargeted sample screening and develop a targeted compound assay, as shown in this study for the presented PRM method.

**Figure 3:**
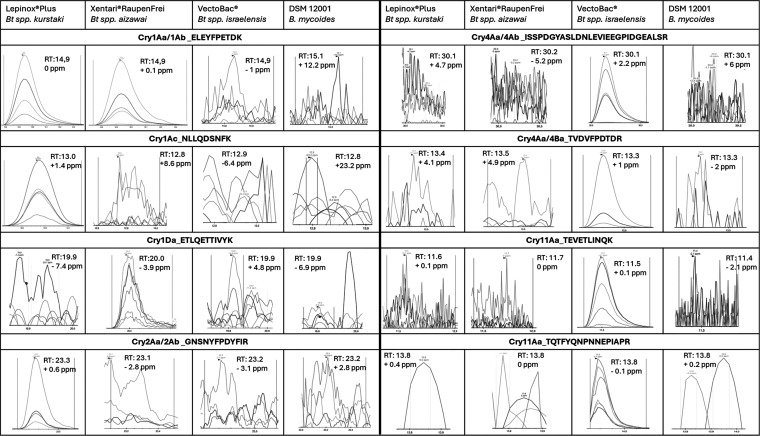
Chromatograms in Skylie for all marker peptides in three *Bt* biopesticides and *B. mycoides* as blank material, with retention time (RT) in minutes and mass accuracy in ppm.

### 3.4 Application of Targeted LC–MS/MS–PRM Method to Treated Plant Material

To assess the applicability of the developed and optimized LC–MS/MS–PRM method to real samples, four commercially available *Bt* biopesticides—Xentari RaupenFrei, LepinoxPlus, Solabiol Zünsler-& and Raupenfrei and VectoBac—were applied to two basil plants each, following the manufacturers’ instructions, as described in Section 2.5 in detail. The identified subspecies and quantified amounts of crystal proteins for each product are presented in Table 3.

**Table 3:** Results for the Application of Four Commercial *Bt* Biopesticides^a^

*Bt* product	Cry1Aa/1Ab [ng/g]	Cry1Ac	Cry1Ca	Cry1 Da	Cry2Aa/2Ab [ng/g]	Cry4Aa/4Ba [ng/g]	Cry11Aa [ng]
LepinoxPlus *Bt* spp. *kurstaki*	0.90 ± 0.04	+	-	-	<LOD	<LOD	<LOD
Solabiol Zünsler-& Raupenfrei *Bt* spp. *kurstaki*	3.10 ± 0.01	+	-	-	<LOD	<LOD	<LOD
Xentari RaupenFrei *Bt* spp. *aizawai*	0.70 ± 0.01	-	+	+	<LOD	<LOD	<LOD
VectoBac *Bt* spp. *israelensis*	<LOD	-	-	-	<LOD	1.2 ± 0.04	0.12 ± 0.00

a *Bt* protein levels (ng/g) were determined in basil plants following the application of products, in accordance with the manufacturer specifications.

In all cases, the identified *Bt* subspecies matched the intended one by the product’s declaration. For the two products containing *Bt* spp. *kurstaki*, LepinoxPlus and Solabiol Zünsler-& Raupenfrei, the Cry1A crystal protein content could be quantified, whereas Cry2Aa/2Ab proteins were below LOD, likely due to the relatively high LOD of 34,36 ng/g. Caballero et al.^24^ reported a balanced contribution between Cry1A and Cry2A proteins in the product DiPelDF. In the case of VectoBac, which is based on *Bt* spp. *israelensis*, higher expression levels of Cry4Aa and Cry4Ab crystal proteins were observed compared to Cry11Aa, which is in line with the findings of Caballero et al.^24^ However, as their study focused on the relative molar composition of different Bt biopesticides, direct comparison with the absolute values obtained in the present study is limited. Furthermore, a comparison of the Cry1A protein content between the two *spp. kurstaki*-based products revealed strain-level differences: strain ABTS-351 (Solabiol Zünsler-& Raupenfrei) exhibited higher Cry1A expression than strain EG-2348 (LepinoxPlus) (Figure 4). Compared to Xentari RaupenFrei, which contains *Bt spp. aizawai*, both *Bt spp. kurstaki* strains demonstrated higher expression levels of Cry1A proteins.

**Figure 4:**
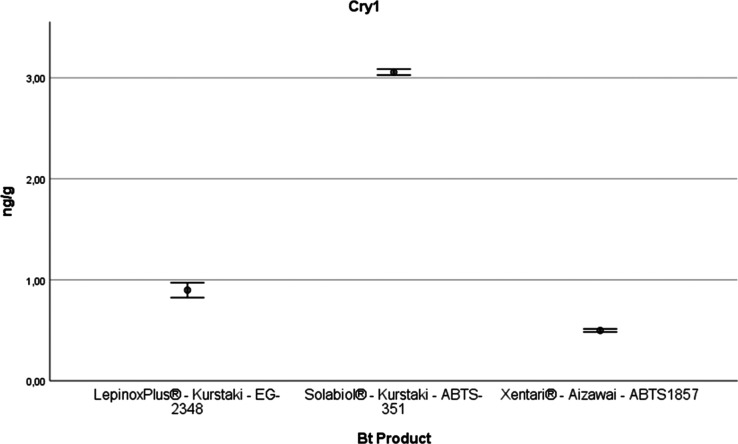
Graph represents the mean values and standard deviation for the content of Cry1 proteins (ng/g) determined by the PRM method for the three *Bt* biopesticides after application to basil plants. Results indicate that different *Bt* strains obtained different amounts of Cry1 proteins.

These findings are consistent with previous reported studies indicating that *cry1*-type genes were more abundant in the isolates of *Bt spp. kurstaki* than in those of *spp. aizawai*.^43,44^ The results confirm the applicability of the developed method for the reliable identification of *Bt* subspecies and the quantification of crystal protein expression in samples treated with commercial *Bt* biopesticides.

This study presented a robust and highly sensitive LC–MS/MS–PRM method for the identification of *Bt* subspecies (*spp. kurstaki*, *spp. aizawai*, and *spp. israelensis*) and the quantification of their pesticidal crystal proteins. Untargeted DIA analysis of various commercial *Bt* biopesticides enabled the initial identification of predominant crystal proteins, which were consistent across isolated microbial samples and treated herbal matrices, with no observable matrix effect. Based on these findings, subspecies-specific and shared marker peptides were selected and validated for both qualitative differentiation and absolute quantification using SIL peptides. The developed PRM method demonstrated high sensitivity, achieving LOD and LOQ in the ng/g range, and high selectivity confirmed through both, in silico and experimental, approaches. The integration of an optimized sample preparation workflow, including protein enrichment, enabled the successful application of the method to *Bt*-treated plant samples. The method reliably distinguished *Bt* subspecies and quantified crystal protein expression in commercial *Bt* biopesticides applied to basil plants. Several conceptual and methodological limitations of the present study should be acknowledged. For qualitative differentiation, the current method is focused on the three authorized and commercially available *Bt* subspecies within the EU. The shared peptide sequence for Cry1 proteins, which occurs across multiple *Bt* subspecies (Table S6), was selected to enable the most comprehensive quantification possible. The modular design of the PRM workflow facilitates an extension of the method to additional *Bt* subspecies or strains through the incorporation of additional marker peptides, but such extension will require the identification and validation of further specific marker peptides. A further limitation of the current method relates matrix diversity. While the application and validation of the method were demonstrated using plant matrices, more complex environmental substrates such as soil or surface water were not included at this stage. These matrices are expected to pose additional analytical challenges due to increased matrix complexity and lower target concentrations; their analysis will require matrix-specific extraction, purification, and enrichment strategies, which represent an important direction for future work. Although no significant matrix effects were observed for the investigated plant samples, this cannot be extrapolated to other environmental compartments without further validation. In addition, the detection limits of certain peptides could be further improved to enable the reliable detection of low-abundance crystal proteins in complex matrices. Immunoassay-based methods currently represent the standard approach for *Bt* Cry protein detection and quantification, as outlined in the Introduction section. Several immunoassays targeting different Cry proteins are available, with the reported detection limits ranging from ng/mL to pg/mL, which are comparable to or, in some cases, more sensitive than the developed LC–MS/MS method.^18^ However, such assays are limited to certain Cry proteins and do not cover, for example, Cry proteins from *Bt spp. israelensis*. Moreover, due to the limited availability of immunoassays, as well as their constraints regarding specificity and multiplexing capability, a direct quantitative comparison was considered of limited relevance for benchmarking the performance of the LC–MS/MS-based approach developed in this study. In addition, the available immunoassays are typically validated only for specific matrices, such as cotton, maize, or water,^18^ and do not cover the plant matrices examined here. Without the dedicated assessment of matrix effects, such comparisons would therefore be of questionable relevance. For these reasons, a direct comparison with immunoassay-based methods was considered beyond the scope of the present work. Nevertheless, future studies would strongly benefit from a systematic comparison with immunoassay-based methods, performed on well-defined matrices and target proteins. Such comparative investigations would help to further position the LC–MS/MS-based approach with respect to sensitivity, selectivity, and practical applicability across a broader range of Cry proteins and sample matrices.

Importantly, this approach contributes to environmental safety assessments by providing quantitative data on crystal protein residues depending on *Bt* subspecies and strains, which is essential for improving exposure characterization in environmental risk assessment frameworks. Nevertheless, quantitative data on Cry protein levels alone are insufficient for a comprehensive environmental safety evaluation. Such data must therefore be integrated with information on insecticidal activity, biological activity, degradation, and persistence of Cry proteins in environmental compartments such as soil and water, as well as their potential effects on nontarget organisms. These aspects were beyond the scope of the present study, which focused on analytical method development, and require further investigation. Beyond environmental considerations, the method also contributes to the quality control of *Bt*-based biopesticides by verifying the presence of declared subspecies used in the formulation and by quantifying the insecticidal protein content. This not only ensures product authenticity but also provides data on protein expression levels for monitoring the batch-to-batch consistency. Moreover, the quantitative results can be used to optimize production processes, improve formulation stability, and enhance the biological efficacy and reliability of *Bt* biopesticides for agricultural applications.

## Supplementary Material



## Data Availability

The raw data and Skyline documents of the targeted method can be found in PanoramaWeb (https://panoramaweb.org/0xo4kt.url). The raw data are available on ProteomXchange with the data set identifier: PXD071216. Reviewer account details: Email: panorama+reviewer384@proteinms.net Password: 2WEi?S$=RMZVvA.

## Notes

Protein content determined by Bradford assay; chromatographic gradient conditions and mass spectrometry settings; data-processing settings and search parameters for PLGS and MASCOT; FASTA file of proteins used for data evaluation of untargeted analyses; calibration curves and data for quantitative marker peptides; BLAST analysis results for marker peptides; and experimental results for selectivity (XLSX)
